# Analysis of Human Blood Plasma Proteome from Ten Healthy Volunteers from Indian Population

**DOI:** 10.1371/journal.pone.0072584

**Published:** 2013-08-20

**Authors:** Poonam Gautam, Sudha C. Nair, Kalidoss Ramamoorthy, Cherukuvada V. Brahmendra Swamy, Ramakrishnan Nagaraj

**Affiliations:** CSIR- Centre for Cellular and Molecular Biology, Hyderabad, India; Indian Institute of Science, India

## Abstract

Analysis of any mammalian plasma proteome is a challenge, particularly by mass spectrometry, due to the presence of albumin and other abundant proteins which can mask the detection of low abundant proteins. As detection of human plasma proteins is valuable in diagnostics, exploring various workflows with minimal fractionation prior to mass spectral analysis, is required in order to study population diversity involving analysis in a large cohort of samples. Here, we used ‘reference plasma sample’, a pool of plasma from 10 healthy individuals from Indian population in the age group of 25–60 yrs including 5 males and 5 females. The 14 abundant proteins were immunodepleted from plasma and then evaluated by three different workflows for proteome analysis using a nanoflow reverse phase liquid chromatography system coupled to a LTQ Orbitrap Velos mass spectrometer. The analysis of reference plasma sample a) without prefractionation, b) after prefractionation at peptide level by strong cation exchange chromatography and c) after prefractionation at protein level by sodium dodecyl sulfate polyacrylamide gel electrophoresis, led to the identification of 194, 251 and 342 proteins respectively. Together, a comprehensive dataset of 517 unique proteins was achieved from all the three workflows, including 271 proteins with high confidence identified by≥2 unique peptides in any of the workflows or identified by single peptide in any of the two workflows. A total of 70 proteins were common in all the three workflows. Some of the proteins were unique to our study and could be specific to Indian population. The high-confidence dataset obtained from our study may be useful for studying the population diversity, in discovery and validation process for biomarker identification.

## Introduction

Determination of the protein constituents of human plasma has been an active area of research for several years [1]. The documentation of a number of proteins that can be detected was highly dependent on the sensitivity of the available detection methods. The list of abundant proteins in the plasma along with their concentration has been documented well before mass spectral methods were deployed [2]. The interest in the protein composition of human plasma has largely stemmed from their relevance in clinical diagnostics [2]–[4]. Mass spectral methods became popular in the analysis of plasma, as it became increasingly possible to detect very low amounts of peptides and proteins [5]–[7]. There have been international collaborative efforts to examine data from different mass spectral instruments and works flows and evolve criteria to arrive at a definitive list of proteins present in the human plasma [8], [9]. Anderson *et al* merged data from four studies reporting in-depth human plasma proteome analysis, including three published experimental datasets using proteomics approach based on different methodologies and fourth dataset drawn from individual published reports on serum or plasma. They reported a non-redundant list of 1,175 gene products, of which 195 proteins appeared in more than one dataset [8]. Another study based on the separation of proteins largely by gel electrophoresis and off-gel electrophoresis, followed by tryptic digestion and analysis using linear ion trap-Orbitrap (LTQ-Orbitrap) and linear quadrupole ion-trap-Fourier transform mass spectrometers, identified a set of 697 proteins with high confidence in the human plasma [10]. Earlier, mass spectral data have been analyzed based on improved algorithm and a list of approximately 1200 proteins have been listed to be present in the plasma [11].

Population proteomics is a recent concept and still emerging. There have been attempts to investigate protein diversity in human population and population specific modification/changes in proteins have been documented [12]–[14]. However, population-specific plasma proteomics has not been investigated as extensively as genomic analysis of populations. The use of standard workflows involving extensive pre-fractionation is one of the important limitations to analyze a larger number of samples to study population diversity or any disease condition in a larger cohort. Hence, in the current study, we have analyzed plasma proteome from Indian population by using strategies that do not involve extensive fractionation. Here, ‘reference plasma sample’, a pool of plasma from 10 healthy individuals, was used for the study. The samples were immunodepleted with 14 most abundant proteins followed by evaluation of three different workflows with minimum pre-fractionation. These include analysis after a) no prefractionation b) prefractionation at peptide level by strong cation exchange (SCX) chromatography and c) prefractionation at protein level by sodium dodecyl sulfate polyacrylamide gel electrophoresis (SDS-PAGE), followed by nanoscale reverse phase liquid chromatography tandem mass spectrometry (nano-RP-LC-MS/MS).

## Materials and Methods

### Sample collection

The Human Ethics Committee at Centre for Cellular and Molecular Biology (CCMB), Hyderabad, India had approved the study. All the blood samples were collected at dispensary of CCMB, Hyderabad, India from the healthy individuals after written informed consent. Blood was collected in EDTA-coated vacutainers from 10 healthy individuals (5 male and 5 female) of Indian origin with age group between 25–60 yrs. The samples were centrifuged at 1500 ×g for 20 min. to separate plasma. Equal volume of plasma from each individual was pooled to get ‘reference plasma sample’. The sample was aliquoted and stored at −80°C until used for further analysis.

### Immunodepletion

Reference plasma sample was immunodepleted using MARS column Hu-14 (4.6×100 mm) on Agilent HPLC-1100 series as per the manufacturer's instruction. Hu-14 column removes albumin, IgG, antitrypsin, IgA, transferrin, haptoglobin, fibrinogen, alpha2-macroglobulin, alpha1-acid glycoprotein, IgM, apolipoprotein Al, apolipoprotein All, complement C3, and transthyretin. The flowthrough fraction was collected (Figure S1A) and desalted using a 5 KDa cutoff spin filters (Agilent Technologies, Santa Clara, CA, USA). Consistency of immunodepletion was confirmed by performing SDS-PAGE analysis (Figure S1B).

### Trypsin digestion

Reference plasma protein sample (500 µg), obtained after immunodepletion, was reduced using dithiothreitol (10 mM DTT) and alkylated using iodoacetamide (50 mM IAA), followed by in-solution digestion with trypsin (Promega, Madison, WI, USA) for 16 h at 37°C overnight. The digest was then lyophilized and stored at −80°C until used. These samples were further analyzed by two of the workflows- a) no prefractionation and b) prefractionation by strong cation exchange (SCX) chromatography. The third workflow involved prefractionation of proteins by SDS-PAGE followed by in-gel trypsin digestion. Proteins separated by SDS-PAGE were subjected to destaining followed by in-gel digestion with trypsin (1∶100 in 25 mM ammonium bicarbonate) (Promega, Madison, WI, USA) for 16 h at 37°C overnight. The peptides were extracted and the digest was lyophilized and stored at −80°C until used.

### Workflows

#### a) No prefractionation

20 µg of tryptic digest was desalted using C18 cartridge (Pierce, Rockford, USA) as per the manufacturer's instructions and 2 µg of the digest was used further for LC-MS/MS analysis.

#### b) Prefractionation at peptide level by SCX chromatographic separation

Tryptic digest from a total of 320 µg protein was resuspended in 1 ml of buffer A [10 mM KH2PO4, 25% (v/v) acetonitrile (ACN), pH 2.9] and separated on a SCX column (Zorbax 300-SCX, 5 µm, 4.5 mm ID × 100 mm, Agilent Technologies, Santa Clara, CA, USA) on Agilent 1100 series HPLC (Figure S2A). The conditions for SCX fractionation include: flow rate 700 µl/min and 40 min gradient- 5 min, 0−5% buffer B (buffer A+1 M KCl); 5 min, 5–10%; 5 min, 10–23%; 5 min, 23–50%; 10 min, 50–100%; 10 min, 100% B. One minute fractions were collected, vacuum-dried and desalted using C18 cartridge (Pierce, Rockford, USA). After desalting, consecutive fractions were pooled to get six fractions with comparable peptide quantities approximated from SCX chromatograms and were subjected to LC-MS/MS analysis.

#### c) Prefractionation at protein level by SDS-PAGE

The Hu-14 immunodepleted and desalted reference plasma sample was fractionated at protein level by 1D-SDS-PAGE. 10 µg of the protein was run on 11 cm, 4–20% Tris-glycine gradient gel (Invitrogen, Carlsbad, CA, USA) in duplicates for half an hour to get a gel with partial run. A total of six bands were excised and cut into small pieces from the Coomassie-stained gel (Figure S2B). The gel pieces were destained with 25 mM ammonium bicarbonate and 50% acetonitrile (ACN), dehydrated in 100% ACN followed by drying with speed vacuum concentrator. In-gel trypsin digestion was performed by rehydration of dried gel pieces with modified sequencing grade trypsin (0.25 µg; Promega, Madison, WI, USA) for 16 h at 37°C. Peptides were extracted with 0.3% triflouroacetic acid (TFA) in 50% acetonitrile (ACN) followed by drying with speed vacuum concentrator. The samples were desalted using Pepclean C18 cartridges (Pierce, Rockford, USA), dried and were further subjected to LC-MS/MS analysis.

### LC-MS/MS analysis

Tryptic digests of samples with no prefractionation, fractionation after SCX (6 fractions) and fractionation after SDS-PAGE (6 fractions) were reconstituted in 0.1% formic acid in 5% acetonitrile (ACN). The samples were analyzed by nano-RP-LC-MS/MS using a nano LC (EASY nLC Proxeon, now Thermo Scientific) connected to LTQ-Velos Orbitrap (Thermo Scientific, Bremen, Germany). A reversed-phase BioBasic C-18 analytical column (5 µm particle size, 300 Å pore size, 75 µm×10 cm) (Thermo Scientific, Bremen, Germany) with picofrit, was used for separation of peptide samples. Tryptic peptides were eluted at a flow rate of 300 nL/min with 60 or 85 min linear gradient of 5–40% (v/v) ACN containing 0.1% (v/v) formic acid.

The voltage applied for ionization was 1.7 kV. The precursor ions MS spectra were acquired in the Orbitrap with resolution of 60,000 at m/z = 400, (mass range 400–2000) with 1×10^6^ accumulated ions. MS/MS was performed for the twenty most intense precursor ions from each MS scan. The peptides were fragmented in the linear ion-trap by collision-induced dissociation with 35% collision energy and the resulting fragment ions were detected at a mass resolution of 15,000 (at m/z 400). Data were acquired using Xcalibur software version 2.1 in data dependent mode. The lock mass option was enabled for accurate measurement in both MS and MS/MS modes. The polydimethylcyclosiloxane ions generated during electrospray from ambient air (m/z, 445.120025) was used for internal calibration in real time [10], [15].

### Bioinformatic Analysis and Protein Identifications

The RAW files were analyzed using both Sequest and Mascot search engines of Proteome Discoverer (Thermo Scientific, Version 1.2) against IPI (International Protein Index) database version 3.75. MS/MS search criterion was as follows: Mass tolerance of 10 ppm for MS and 0.25 Da for MS/MS mode, trypsin as the enzyme with 1 missed cleavage allowed, carbamido methylation of cysteine as static and methionine oxidation as dynamic modifications respectively. For Sequest search analysis Xcorr was set at 1.9 (1^+^), 2.2 (2^+^), and 2.3 (3^+^) and for Mascot search cut off score was set at ≥30. High confidence peptides were used for protein identifications by setting a target false discovery rate (FDR) threshold of 1% at the peptide level. Only unique peptides with high confidence and rank 1 were used for protein identifications. Proteins identified with ≥2 peptides in any workflow or by a single peptide identified in any of the two workflows was considered to be identified with high confidence. Only unique peptides with high confidence and rank 1 were used for protein identifications. Biological function and localization of proteins were obtained from Gene Ontology database (http://www.geneontology.org). Mass spectrometry raw data are available with PG and RN.

## Results and Discussion

**Figure 1:**
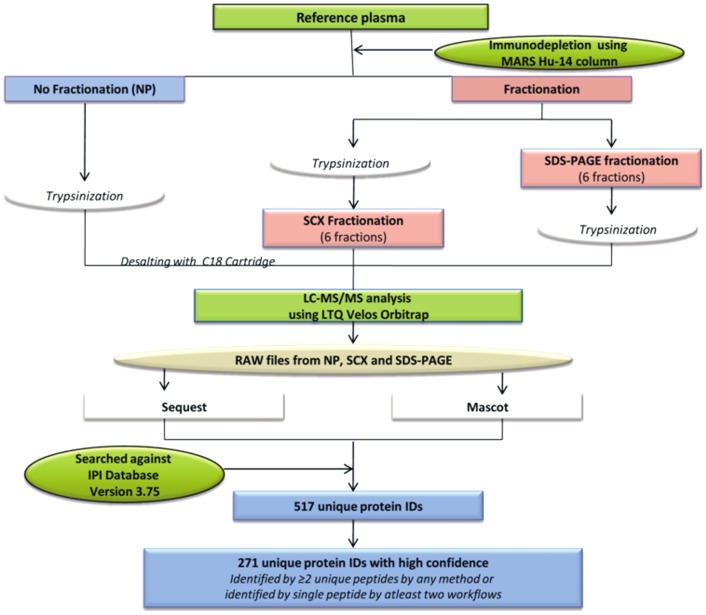
Experimental overview and bioinformatic analysis to study plasma proteome. Reference plasma sample was prepared by pooling equal volumes of plasma from 10 healthy individuals of either sexes and age group of 25–60 years. The sample was immunodepleted with 14 abundant proteins and analyzed using three different workflows- a) no prefractionation b) prefractionation at peptide level by strong cation exchange (SCX) chromatography and c) prefractionation at protein level by sodium dodecyl sulfate polyacrylamide gel electrophoresis (SDS-PAGE), using nano LC-MS/MS approach for in-depth human plasma proteome analysis. The analysis led to the identification of a total of 517 unique proteins identified from all the three workflows. A total of 271 proteins were identified with high confidence i.e. identified with≥2 unique peptides or by a single peptide identified in any of the two workflows.

The current study has attempted to evaluate the workflows involving minimal prefractionation that may be employed for studying population proteomics. As an initial effort, we used ‘reference plasma sample’, a pool of plasma from 10 healthy individuals with various age groups and gender, followed by immunodepletion of 14 most abundant proteins to improve the identification of low abundant proteins. The bound and flowthrough fractions were separated clearly (Figure S1A). We explored three different workflows and analyzed the plasma sample after a) no prefractionation b) prefractionation of the tryptic peptides using SCX chromatography and c) prefractionation of proteins using SDS-PAGE. The experimental overview and bioinformatic analysis is shown in the Figure 1.

Mass spectral data obtained from each workflow was analyzed separately using Sequest or Mascot search node. The individual data files from analysis of SCX fractions (6 fractions) or by SDS-PAGE (6 bands) from Sequest or Mascot search node were merged for protein identifications (Table S1A-F). Data obtained after Mascot or Sequest search analysis for each work flow were then merged, redundant proteins were removed and a final list of proteins was obtained. The number of unique proteins identified after no prefractionation, prefractionation by SCX and prefractionation by SDS-PAGE was 194, 251 and 342, of which 56, 112 and 201 proteins were specific to each workflow respectively. The data from each workflow are summarized in Table S2A-C. After combining the data from all the three workflows, a total of 517 unique or non-redundant proteins was obtained and listed in Table S3. Of these, 271 proteins were identified with ≥2 unique peptides or by a single peptide identified in any of the two workflows. The remaining proteins were identified by single peptide in any of the workflows. We compared the proteins identified with high confidence (n = 271) in our study with the dataset of 697 and 1,175 proteins reported by Schenk *et al* [10] and Anderson *et al* [8] respectively. A total of 121 and 82 proteins were found to be common with these datasets. Overall, a total of 72 proteins were common between three datasets i.e. dataset from the current study, Schenk *et al* and Anderson *et al* study (see Table S3). In our data, although several proteins were initially listed as ‘uncharacterized’ by searching against IPI database, subsequent search in UniProt database revealed their IDs that are indicated in the Table S3.

**Figure 2:**
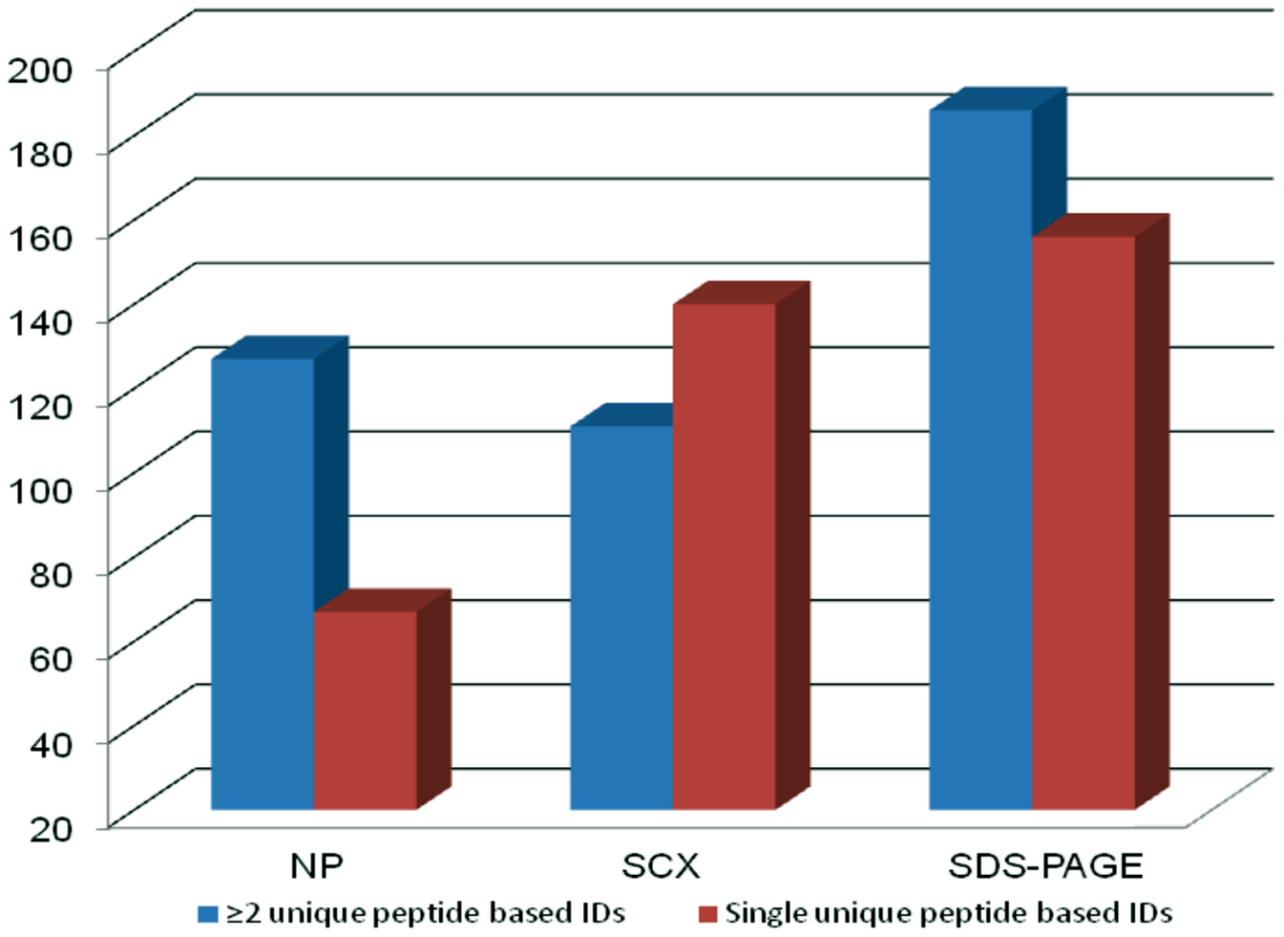
Histogram showing total number of proteins identified with≥2 unique peptides and single peptide in three different workflows. NP- No prefractionation; SCX- Strong cation exchange chromatography; SDS-PAGE- sodium dodecyl sulfate polyacrylamide gel electrophoresis.

**Figure 3:**
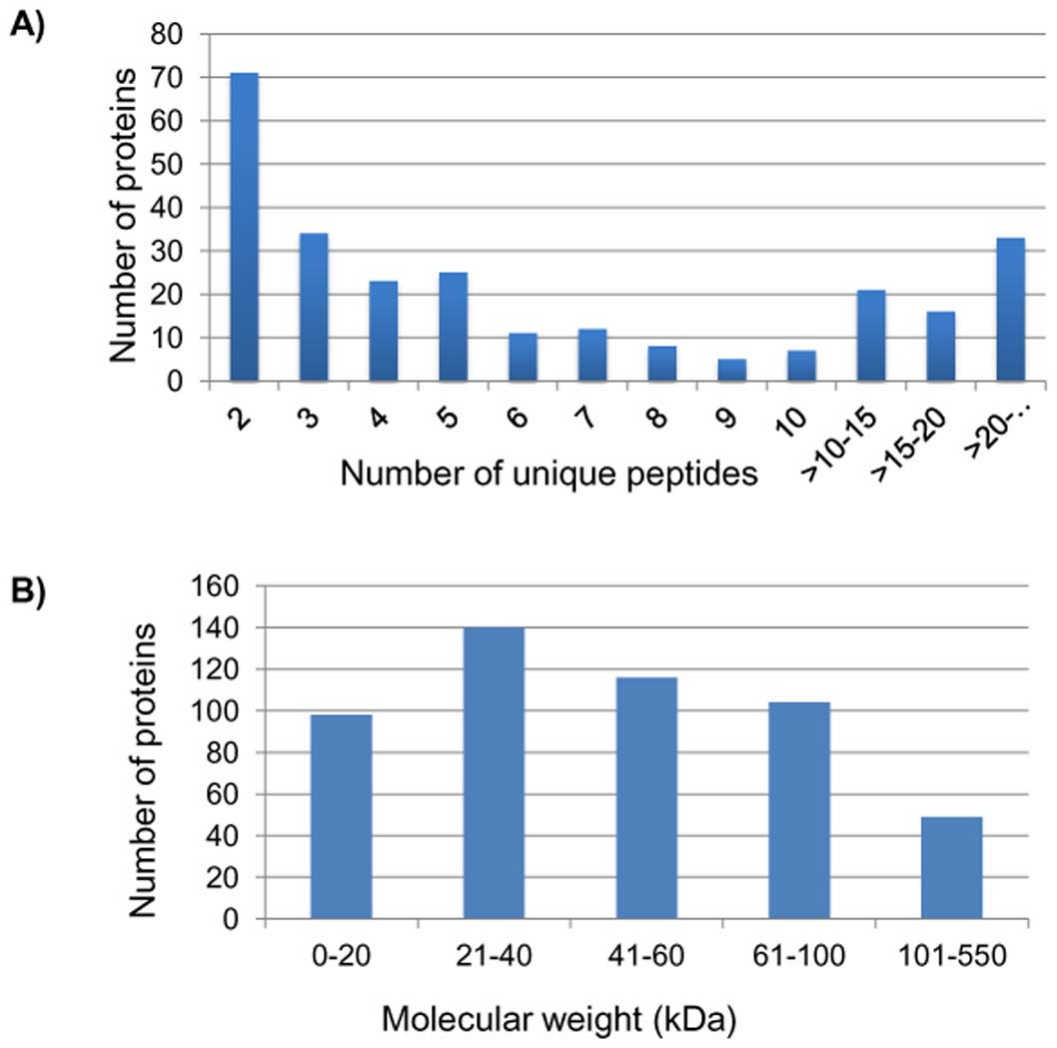
Histograms showing the number of proteins identified with unique peptides and molecular weight distribution of proteins identified in the study. (A) Number of proteins identified based on 2 or more unique peptides. Approximately 70 proteins were identified with 2 peptides. Several proteins were identified with≥10 peptides. (B) Molecular Weight distribution of the identified proteins. Maximum number of proteins identified had molecular weights in the range 21–40 kDa.

**Figure 4:**
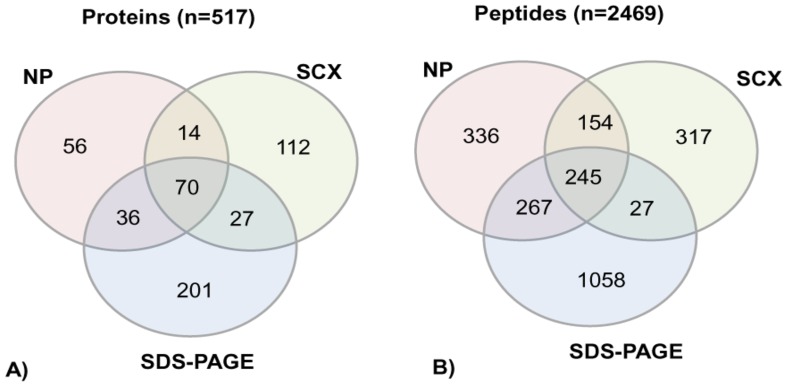
Venn diagram showing consensus of unique proteins and peptides identified in three different workflows. A total of 70 proteins (A) and 245 peptides (B) were identified in all the three workflows. NP- No prefractionation; SCX- Strong cation exchange chromatography; SDS-PAGE- sodium dodecyl sulfate polyacrylamide gel electrophoresis.

**Figure 5:**
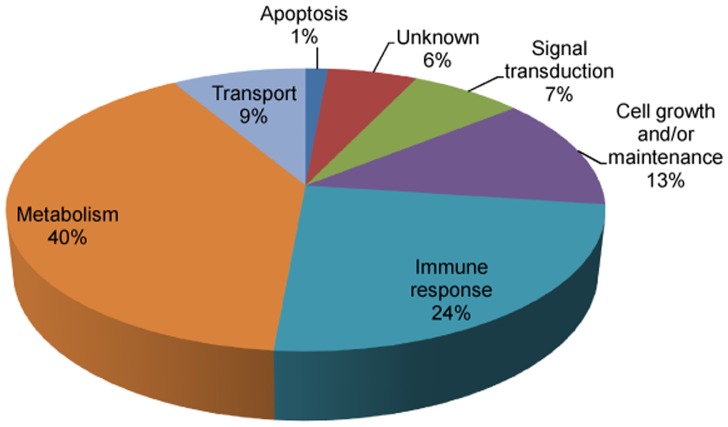
Pie chart showing biological processes of 70 proteins identified with high confidence in all the three workflows.

The proteins identified by single and ≥2 peptides in three different work flows are shown in Figure 2. Interestingly, the workflows that include no prefractionation and prefractionation at protein level before digestion yielded greater number of protein IDs with ≥2 unique peptides as compared to single peptide IDs. Only in the workflow, in which tryptic peptides were prefractionated on a SCX column, the number of IDs based on single peptide was more. The number of proteins versus number of unique peptides and molecular weight are shown in Figure 3A and 3B. Most of the proteins were identified with ≥2 peptides. Seventy seven proteins were identified with ≥10 peptides. While maximum number of identified proteins had molecular weights in the range 21–40 kDa, a large number of high molecular weight proteins have also been identified. The distribution is similar to the analysis by Schenk *et al* [10]. Degree of consensus of protein IDs and peptides identified in three workflows is represented as Venn diagram (Figure 4). A total of 70 proteins and 245 unique peptides were identified in all the three workflows. These proteins are listed in Table 1. More than 70% of the proteins match with the plasma proteins identified by Schenk *et al* [10] and/or Anderson *et al* [8] and have been indicated in Table 1. The cellular localization of these proteins reveals that most of them are extracellular. These proteins participate in various biological processes related to metabolism, immune response, cell growth and/or maintenance, transport and signal transduction as shown in Figure 5. Some of the important biological processes and proteins include cell communication and signal transduction (pigment epithelium-derived factor, insulin-like growth factor-binding protein complex acid labile subunit and retinol binding protein 4, plasma); cell growth and/or maintenance (thrombospondin-1, afamin and isoform C of fibulin-1); and transport (serum amyloid A-4 protein, hemopexin, apolipoprotein B-100, apolipoprotein E, vitamin D-binding protein isoform 1 precursor).

**Table 1:** Plasma proteins identified with high confidence in all the three workflows.

Accession	Gene Symbol	Description	Unique peptides	MW [kDa]	Calc. pI	Localization	Comparison with Schenk et al. (Ref 10)^a^	Comparison with Anderson et al (Ref 8) a	Biological Process
IPI:IPI00006114.5	SERPINF1	Pigment epithelium-derived factor	13	46.3	6.38	Extracellular	+	+	Cell communication; Signal transduction
IPI:IPI00007199.4	SERPINA10	Protein Z-dependent protease inhibitor	7	55.1	7.64	Extracellular	+	+	Protein metabolism
IPI:IPI00009865.4	KRT10	Keratin, type I cytoskeletal 10	24	58.8	5.21	Cytoplasm	+	−	Cell growth and/or maintenance
IPI:IPI00010295.1	CPN1	Carboxypeptidase N catalytic chain	8	52.3	7.34	Extracellular	+	+	Protein metabolism
IPI:IPI00011252.1	C8A	Complement component C8 alpha chain	13	65.1	6.47	Extracellular	+	+	Immune response
IPI:IPI00017601.1	CP	Ceruloplasmin	50	122.1	5.72	Extracellular	+	+	Metabolism; Energy pathways
IPI:IPI00019399.2	SAA4	Serum amyloid A-4 protein	2	14.7	9.07	Extracellular	+	+	Transport
IPI:IPI00019568.1	F2	Prothrombin (Fragment)	32	70	5.9	Extracellular	+	+	Protein metabolism
IPI:IPI00019576.1	F10	Coagulation factor X	4	54.7	5.94	Extracellular	+	+	Protein metabolism
IPI:IPI00019580.1	PLG	Plasminogen	45	90.5	7.24	Extracellular	+	+	Protein metabolism
IPI:IPI00019591.2	CFB	cDNA FLJ55673, highly similar to Complement factor B	41	140.9	7.18	Extracellular	+	−	Immune response
IPI:IPI00019943.1	AFM	Afamin	22	69	5.9	Extracellular	+	−	Cell growth and/or maintenance
IPI:IPI00020996.5	IGFALS	Insulin-like growth factor-binding protein complex acid labile subunit	12	66	6.79	Extracellular	+	+	Cell communication; Signal transduction
IPI:IPI00021842.1	APOE	Apolipoprotein E	18	36.1	5.73	Extracellular	+	+	Transport
IPI:IPI00022229.2	APOB	Apolipoprotein B-100	190	515.3	7.05	Extracellular	+	+	Transport
IPI:IPI00022371.1	HRG	Histidine-rich glycoprotein	16	59.5	7.5	Extracellular	+	+	Apoptosis
IPI:IPI00022391.1	APCS	Serum amyloid P-component	11	25.4	6.54	Extracellular	+	+	Protein metabolism
IPI:IPI00022395.1	C9	Complement component C9	18	63.1	5.59	Extracellular	+	+	Immune response
IPI:IPI00022417.4	LRG1	Leucine-rich alpha-2-glycoprotein	13	38.2	6.95	Extracellular	+	+	Biological_process unknown
IPI:IPI00022488.1	HPX	Hemopexin	24	51.6	7.02	Extracellular	+	+	Transport
IPI:IPI00022895.7	A1BG	Alpha-1B-glycoprotein	13	54.2	5.87	Extracellular	−	−	Biological_process unknown
IPI:IPI00022895.8	A1BG	Isoform 1 of Alpha-1B-glycoprotein	15	54.2	5.86	Extracellular	+	−	Biological_process unknown
IPI:IPI00026199.2	GPX3	Glutathione peroxidase 3	5	25.5	8.05	Extracellular	+	+	Metabolism; Energy pathways
IPI:IPI00027827.2	SOD3	Extracellular superoxide dismutase [Cu-Zn]	4	25.8	6.61	Extracellular	+	−	Metabolism; Energy pathways
IPI:IPI00029193.1	HGFAC	Hepatocyte growth factor activator	7	70.6	7.24	Extracellular	+	+	Protein metabolism
IPI:IPI00029717.1	FGA	Isoform 2 of Fibrinogen alpha chain	23	69.7	8.06	Extracellular	−	−	Protein metabolism
IPI:IPI00029739.5	CFH	Isoform 1 of Complement factor H	49	139	6.61	Extracellular	+	−	Immune response
IPI:IPI00032179.3	SERPINC1	Antithrombin-III	31	52.6	6.71	Extracellular	+	+	Protein metabolism
IPI:IPI00032220.3	AGT	Angiotensinogen	13	53.1	6.32	Extracellular	+	+	Cell communication; Signal transduction
IPI:IPI00032291.2	C5	Complement C5	44	188.2	6.52	Extracellular	+	+	Immune response
IPI:IPI00032293.1	CST3	Cystatin-C	1	15.8	8.75	Extracellular	+	+	Protein metabolism
IPI:IPI00032311.4	LBP	Lipopolysaccharide-binding protein	5	53.3	6.7	Extracellular	+	+	Immune response
IPI:IPI00163207.1	PGLYRP2	Isoform 1 of N-acetylmuramoyl-L-alanine amidase	13	62.2	7.55	Extracellular	+	−	Immune response
IPI:IPI00166729.4	AZGP1	Zinc-alpha-2-glycoprotein	21	34.2	6.05	Extracellular	+	+	Immune response
IPI:IPI00215983.3	CA1	Carbonic anhydrase 1	6	28.9	7.12	Cytoplasm	+	−	Metabolism; Energy pathways
IPI:IPI00218413.2	BTD	Biotinidase	5	61.1	6.25	Extracellular	+	+	Metabolism; Energy pathways
IPI:IPI00218732.4	PON1	Serum paraoxonase/arylesterase 1	8	39.7	5.22	Extracellular	+	+	Metabolism; Energy pathways
IPI:IPI00219713.1	FGG	Isoform Gamma-A of Fibrinogen gamma chain	20	49.5	6.09	Extracellular	−	−	Protein metabolism
IPI:IPI00292530.1	ITIH1	Inter-alpha-trypsin inhibitor heavy chain H1	29	101.3	6.79	Extracellular	+	+	Protein metabolism
IPI:IPI00292946.1	SERPINA7	Thyroxine-binding globulin	11	46.3	6.3	Extracellular	+	+	Protein metabolism
IPI:IPI00294395.1	C8B	Complement component C8 beta chain	16	67	8.13	Extracellular	+	+	Immune response
IPI:IPI00296099.6	THBS1	Thrombospondin-1	7	129.3	4.94	Extracellular	+	+	Cell growth and/or maintenance
IPI:IPI00296537.4	FBLN1	Isoform C of Fibulin-1	12	74.4	5.24	Extracellular	+	−	Cell growth and/or maintenance
IPI:IPI00296608.6	C7	Complement component C7	21	93.5	6.48	Extracellular	+	+	Immune response
IPI:IPI00298497.3	FGB	Fibrinogen beta chain	24	55.9	8.27	Extracellular	+	+	Protein metabolism
IPI:IPI00298971.1	VTN	Vitronectin	12	54.3	5.8	Extracellular	+	+	Cell growth and/or maintenance
IPI:IPI00299503.2	GPLD1	Isoform 1 of Phosphatidylinositol-glycan-specific phospholipase D	12	92.3	6.37	Extracellular	+	−	Cell communication; Signal transduction
IPI:IPI00304273.2	APOA4	Apolipoprotein A-IV	38	45.4	5.38	Extracellular	+	+	Transport
IPI:IPI00306378.5	MASP2	Isoform 2 of Mannan-binding lectin serine protease 2	1	20.6	5.96	Extracellular	−	−	Protein metabolism
IPI:IPI00328609.3	SERPINA4	Kallistatin	16	48.5	7.75	Extracellular	−	−	Protein metabolism
IPI:IPI00418163.3	C4B	Complement C4-B preproprotein	64	192.6	7.27	Extracellular	+	−	Immune response
IPI:IPI00478809.4	F5	Coagulation factor V	23	251.5	6.05	Extracellular	−	+	Protein metabolism
IPI:IPI00479723.5	FN1	Isoform 10 of Fibronectin	22	239.5	5.88	Extracellular	−	−	Cell growth and/or maintenance
IPI:IPI00480192.1	RTN4RL2	Retinol binding protein 4, plasma	10	22.9	6.09	Plasma membrane	−	−	Cell communication; Signal transduction
IPI:IPI00555812.5	GC	Vitamin D-binding protein isoform 1 precursor	34	52.9	5.45	Extracellular	+	−	Transport
IPI:IPI00556459.2	SERPING1	cDNA FLJ58564, highly similar to Plasma protease C1 inhibitor	14	49.7	6.54	Extracellular	−	−	Protein metabolism
IPI:IPI00643948.2	C1QB	Complement component 1, q subcomponent, B chain	4	24.4	9.16	Extracellular	−	−	Immune response
IPI:IPI00656111.1	PRG4	Isoform E of Proteoglycan 4	6	102.4	9.63	Extracellular	−	−	Cell growth and/or maintenance
IPI:IPI00794777.1	PRDX2	Uncharacterized protein	2	15.1	6.13	Plasma membrane	−	−	Metabolism; Energy pathways
IPI:IPI00795633.1	CLU	CLU	18	52.3	6.38	Extracellular	−	−	Immune response
IPI:IPI00815947.1	SERPING1	Truncated beta-globin (Fragment)	2	4.5	9.47	Extracellular	−	−	Protein metabolism
IPI:IPI00847635.1	SERPINA3	Isoform 1 of Alpha-1-antichymotrypsin	23	47.6	5.52	Extracellular	−	−	Protein metabolism
IPI:IPI00879573.1	SERPIND1	Heparin cofactor 2	22	57	6.9	Extracellular	−	+	Protein metabolism
IPI:IPI00879709.3	C6	Complement component 6 precursor	28	105.7	6.79	Extracellular	−	−	Immune response
IPI:IPI00887739.3	C3	complement C3-like, partial	7	144.7	7.52	Extracellular	−	−	Immune response
IPI:IPI00910249.1	COMP	cDNA FLJ59562, highly similar to Cartilage oligomeric matrix protein	2	77.2	4.53	Extracellular	−	−	Cell growth and/or maintenance
IPI:IPI00922213.2	FN1	cDNA FLJ53292, highly similar to Homo sapiens fibronectin 1 (FN1), transcript variant 5, mRNA	4	111.2	6.21	Extracellular	−	−	Cell growth and/or maintenance
IPI:IPI00939169.1	ATRN	Isoform 3 of Attractin	4	133.6	6.98	Extracellular	−	−	Immune response
IPI:IPI00966295.1		Light chain of factor I	16	65	7.5		−	−	
IPI:IPI00967977.2	CD14	Conserved hypothetical protein	5	31.7	6.29	Extracellular	−	−	Immune response

a The symbols+and–indicate presence and absence of a protein when compared our dataset with the dataset by Schenk *et al*. [10] and Anderson *et al.* [8] study.

Among these, a number of proteins identified here have been implicated in various disease processes. Pigment epithelium-derived factor, a glycoprotein that belongs to the superfamily of serine protease inhibitors, has been shown to be a potent inhibitor of angiogenesis in the mammalian eye, and is involved in the pathogenesis of angiogenic eye diseases [16], [17]. Fibulin-l is reported to be involved in the spread of ovarian cancer in the peritoneal cavity and/or in distal metastases [18]. Plasma retinol binding protein 4 has been reported as a potential biomarker of nephropathy and cardiovascular disease in type 2 diabetic subjects [19]. Apolipoprotein B, involved in transport, has been implicated in cardiovascular diseases [20], [21]. Apolipoprotein E (APOE) is largely produced by glial cells and its genotype is reported to be one of the major genetic risk factor for Alzheimer disease [22]. Reduced conversion of vitamin D-binding protein to a macrophage activation factor has been reported to be valuable to determine risk of disease extension in juvenile idiopathic arthritis (JIA) patients [23]. The presence of such proteins in our datasets suggests the possibility to study population diversity, in discovery and validation process for biomarker identification, in a larger cohort.

Interestingly, keratin IDs account for approximately 3.5% of total proteins in the list of 517 proteins. We also identified keratin isoform 10, a marker for poor prognosis in hepatocellular carcinoma patients after resection [24]. A large number of single peptide-based hits had Mascot and Sequest scores generally considered as acceptable. The number of ions obtained in MS/MS was also ≥50% of the theoretically possible fragmentation. They were also included in the comprehensive list of proteins in Table S3. By employing the strategy described in this paper, we have been able to identify a large number of proteins present in the human plasma. The HPLC method of separation of abundant proteins appears to be effective, as the 14 depleted proteins were not observed in the unbound fraction. We have detected a large number of ‘classical plasma proteins’ in addition to some proteins leaked from tissue. Human plasma is dynamic and it is unlikely there is an absolute number of proteins. The levels may also vary depending on several factors. The concentration range of proteins detected was from 9–20 µM (hemopexin) to 0.04–0.08 µM (cystatin C) a dynamic range of 10^3^. A total of 140 out of 271 proteins identified with high confidence that did not match to the proteins reported by Schenk *et al* and Anderson *et al*, marked by (-) sign in Tables 1 and S3, are unique to this study and could be specific to Indian population. There have been attempts to investigate protein diversity in human population implicated in cancer, with various modifications observed in abundant proteins [12], [25]. We have noted the presence of some of the abundant proteins such as apolipoprotein CII, apolipoprotein CIII, apolipoprotein E, antithrombin III, and cystatin C even when the sample was analyzed without any prefractionation was analyzed. Hence, it should be possible to do directed proteomics to examine post-translational modification or changes in peptide sequence without extensive fractionation of plasma proteins after depletion of abundant proteins using the power of Orbitrap Velos mass spectrometer.

The current study was designed to evaluate the workflows employing minimal pre-fractionation towards the goal of population proteomics and not for in-depth analysis to identify a large dataset of proteins from plasma, which may require extensive fractionation. Here, we have used three different workflows to identify proteins in the pooled plasma of individuals from Indian population and could achieve a comprehensive dataset of 517 unique proteins with 271 proteins identified with high confidence. Of these, 70 proteins were identified in all the workflows and could be targeted in our future studies, considering that ‘population proteomics’ study may focus on a small/targeted dataset analyzed in larger cohort of samples. Even without fractionation of plasma depleted of abundant proteins, a large number of proteins were detected. The strategies employed here can be applied for quantitative analysis such as iTRAQ labeling of proteins/peptides or label free quantitation. Though, the number of proteins identified in our study is relatively less, we believe that the protein dataset identified with high confidence in our study include functionally relevant proteins and would be useful to address population diversity for validation process, where a large cohort is required to establish the outcome.

## Supporting Information

Figure S1 Immunodepletion of reference plasma proteins using MARS column Hu-14 using high pressure liquid chromatography and SDS-PAGE analysis of the flowthrough fraction. (A) Hu-14 column removes 14 most abundant plasma proteins. The bound and flowthrough fraction were clearly separated. (B) Consistency of immunodepletion was confirmed by SDS-PAGE analysis of the flowthrough fraction. FT- Flowthrough
(TIF)

Figure S2 Prefractionation of immunodepleted reference plasma proteins at peptide and protein level using SCX chromatography and SDS-PAGE respectively. (A) SCX chromatogram showing fractionation at peptide level. A total of 320 µg protein was digested with trypsin and peptides were fractionated using SCX column on Agilent 1100 series HPLC. After desalting, consecutive fractions were pooled to get six fractions with comparable peptide quantities approximated from SCX chromatograms and were subjected to LC-MS/MS analysis (see methods). (B) SDS-PAGE showing prefractionation at protein level. A total of 10 µg of the protein was separated using SDS-PAGE for half an hour to get a partial run. A total of six bands were excised and subjected to in-gel digestion. The samples were desalted and were further subjected to LC-MS/MS analysis (see methods). SCX- Strong cation exchange chromatography; SDS-PAGE- sodium dodecyl sulfate polyacrylamide gel electrophoresis
(TIF)

Table S1 (A) List of proteins identified from sample with no prefractionation after search analysis using Sequest search node. The corresponding unique peptides, PSM, molecular weight and pI are shown in the table. (B) List of proteins identified from sample with no prefractionation after search analysis using Mascot search node. The corresponding unique peptides, PSM, molecular weight and pI are shown in the table. (C) List of proteins identified after SCX fractionation and search analysis by Sequest search node. The corresponding unique peptides, PSM, molecular weight and pI are shown in the table. (D) List of proteins identified after SCX fractionation and search analysis by Mascot search node. The corresponding unique peptides, PSM, molecular weight and pI are shown in the table. (E) List of proteins identified after fractionation by SDS-PAGE and search analysis by Sequest search node. The corresponding unique peptides, PSM, molecular weight and pI are shown in the table. (F) List of proteins identified after fractionation by SDS-PAGE and search analysis by Sequest search node. The corresponding unique peptides, PSM, molecular weight and pI are shown in the table.
(XLS)

Table S2 (A) List of proteins after merging the data obtained from Sequest and Mascot search node from the sample with no prefractionation. (B) List of proteins after merging the data obtained from Sequest and Mascot search node from the sample after prefractionation at peptide level by SCX chromatography. (C) List of proteins after merging the data obtained from Sequest and Mascot search node from the sample after prefractionation at protein level by SDS-PAGE.
(XLS)

Table S3 A comprehensive dataset of 517 unique proteins identified from all the three workflows. A total of 271 proteins were identified with≥2 unique peptides or by a single peptide identified in any of the two workflows. Another 246 proteins were identified by single peptide and any one of the workflows. The workflow(s) in which a protein was identified is shown in the table. The dataset of 271 proteins identified with high confidence in our dataset was compared with published plasma protein dataset by Schenk et al [Ref. 10], and Anderson et al [Ref 8] and 121 and 82 proteins respectively were found to be matching to our dataset.
(XLS)
