# Supplementary figures and images for: Analysis of Human Blood Plasma Proteome from Ten Healthy Volunteers from Indian Population
Source: PLoS One. 2013 Aug 20;8(8):e72584. doi: 10.1371/journal.pone.0072584 (PMC3748081; doi:10.1371/journal.pone.0072584)

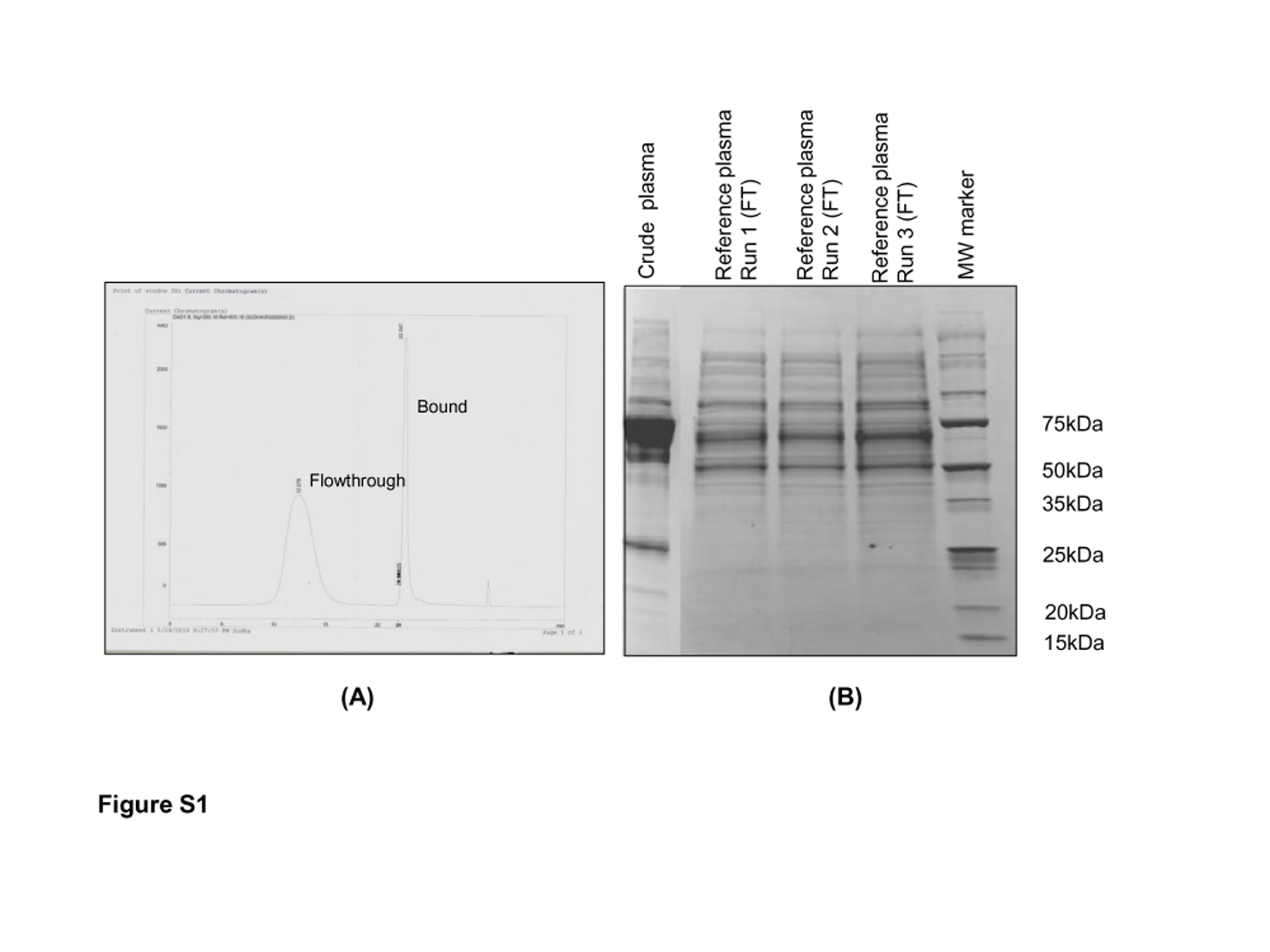

Supplement: Figure S1 — Immunodepletion of reference plasma proteins using MARS column Hu-14 using high pressure liquid chromatography and SDS-PAGE analysis of the flowthrough fraction. (A) Hu-14 column removes 14 most abundant plasma proteins. The bound and flowthrough fraction were clearly separated. (B) Consistency of immunodepletion was confirmed by SDS-PAGE analysis of the flowthrough fraction. FT- Flowthrough (TIF) [file pone.0072584.s001.tif]

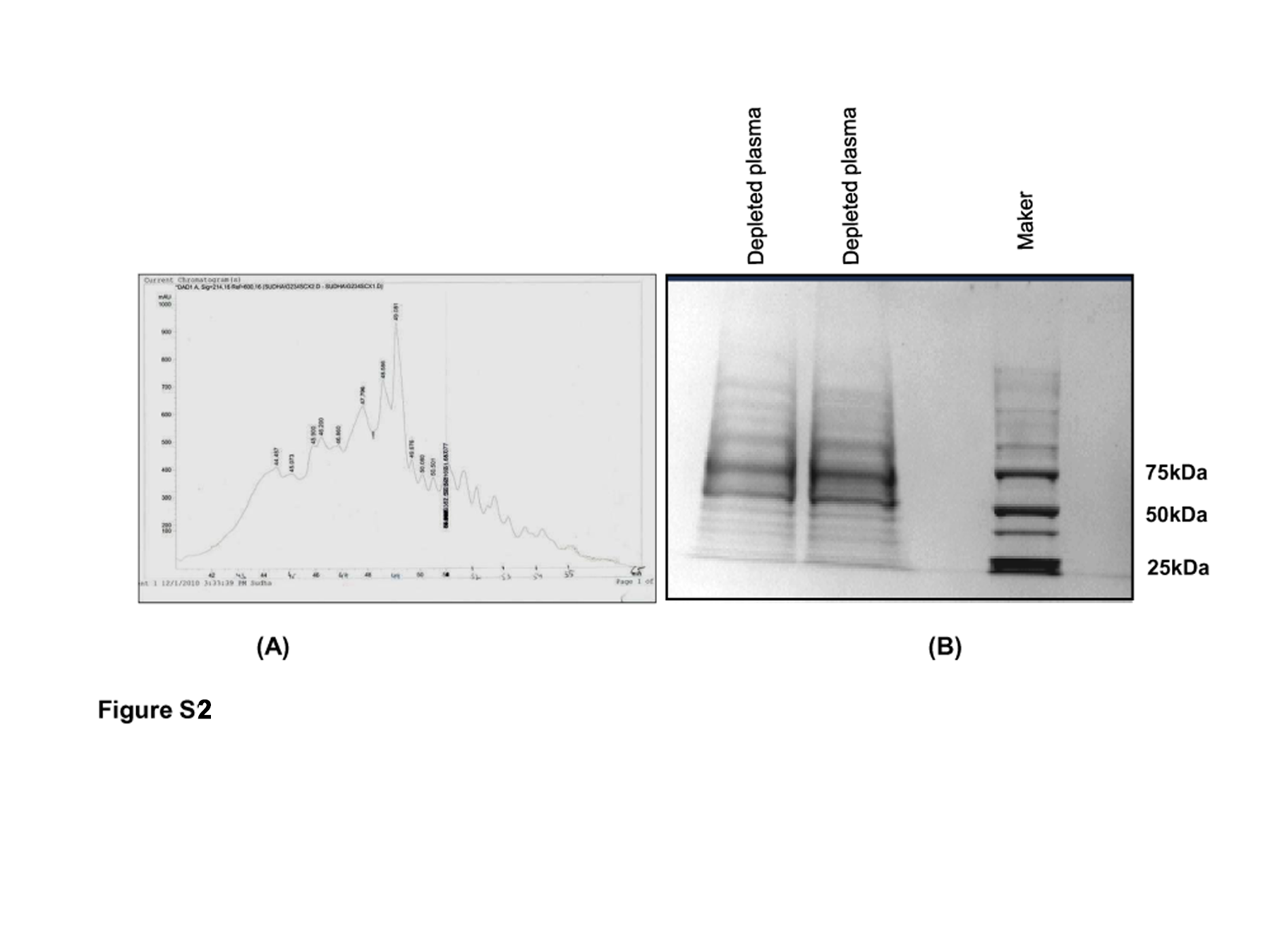

Supplement: Figure S2 — Prefractionation of immunodepleted reference plasma proteins at peptide and protein level using SCX chromatography and SDS-PAGE respectively. (A) SCX chromatogram showing fractionation at peptide level. A total of 320 µg protein was digested with trypsin and peptides were fractionated using SCX column on Agilent 1100 series HPLC. After desalting, consecutive fractions were pooled to get six fractions with comparable peptide quantities approximated from SCX chromatograms and were subjected to LC-MS/MS analysis (see methods). (B) SDS-PAGE showing prefractionation at protein level. A total of 10 µg of the protein was separated using SDS-PAGE for half an hour to get a partial run. A total of six bands were excised and subjected to in-gel digestion. The samples were desalted and were further subjected to LC-MS/MS analysis (see methods). SCX- Strong cation exchange chromatography; SDS-PAGE- sodium dodecyl sulfate polyacrylamide gel electrophoresis (TIF) [file pone.0072584.s002.tif]
